# Neonatal pneumococcal conjugate vaccine immunization primes T cells for preferential Th2 cytokine expression: A randomized controlled trial in Papua New Guinea

**DOI:** 10.1016/j.vaccine.2008.12.046

**Published:** 2009-02-25

**Authors:** Anita. H.J. van den Biggelaar, Peter C. Richmond, William S. Pomat, Suparat Phuanukoonnon, Marie A. Nadal-Sims, Catherine J. Devitt, Peter M. Siba, Deborah Lehmann, Patrick G. Holt

**Affiliations:** a Telethon Institute for Child Health Research, Centre for Child Health Research, University of Western Australia, PO Box 855, West Perth, WA 6872, Australia; b School of Paediatrics and Child Health, University of Western Australia, GPO Box D184, Perth, WA 6840, Australia; c Papua New Guinea Institute of Medical Research, PO Box 60, Goroka, EHP 441, Papua New Guinea

**Keywords:** Pneumococcal conjugate vaccine, Neonatal, T cells

## Abstract

The effects of neonatal immunization with 7-valent pneumococcal conjugate vaccine (7vPCV) on development of T-cell memory and general immune maturation were studied in a cohort of Papua New Guinean newborns. Neonatal 7vPCV priming (followed by a dose at 1 and 2 months of age) was associated with enhanced Th2, but not Th1, cytokine responses to CRM_197_ compared to 7vPCV at 1 and 2 months of age only. T cell responses to non-7vPCV vaccine antigens were similar in all groups, but TLR-mediated IL-6 and IL-10 responses were enhanced in 7vPCV vaccinated compared to controls. Neonatal 7vPCV vaccination primes T cell responses with a polarization towards Th2 with no bystander effects on other T cell responses.

## 1 Introduction

Every year about 1 million children under 5 years of age die of pneumococcal pneumonia, meningitis or sepsis, mostly in developing countries [1]. Immunization with a 7-valent or 9-valent pneumococcal conjugate vaccine (7vPCV or 9vPCV) has been found to be efficacious in reducing severe morbidity and mortality in industrialized and resource-poor countries when given at the age of 6 weeks or older [2–5]. However, *Streptococcus pneumoniae* remains an important cause of serious morbidity and mortality in children in the first 3 months of life [6–8]. In the highlands of Papua New Guinea (PNG), where this study was performed, the mortality rate for pneumonia in infants is 25/1000/year [9] and *S. pneumoniae* accounts for almost half of all bacteraemic pneumonia (as well as meningitis) cases [10,11]. Accelerated immunization schedules, including neonatal vaccination, should therefore be considered in high-risk populations to induce the earliest possible protection against invasive pneumococcal disease.

In contrast to pneumococcal polysaccharide vaccines, conjugate vaccines can elicit memory responses, even in young children. The memory T cell response that is induced against the carrier protein (in 7vPCV this is a non-toxic diphtheria toxin mutant protein, CRM_197_) is a key factor in the induction of long term protection by providing help to polysaccharide-specific memory B cell responses [12–14]. In human newborns and young infants T cell responses are immature and characterized by deficient T helper 1 (Th1) responses [15–18] and a reduced capacity to induce T cell memory [19]. Therefore neonatal conjugate vaccination could fail to provide long-term protection, as shown in neonatal mice that were unsuccessful in producing T cell responses upon vaccination [20], or may result in the induction of tolerance to subsequent vaccination or infection. However, under appropriate priming conditions, neonatal T cells do have the intrinsic capacity to generate memory T(h1) responses as has been shown following neonatal BCG vaccination [21,22]. With respect to conjugate vaccines, the effect of neonatal immunization on T-cell responses has not been evaluated. Studies that measured antibody responses following neonatal immunization with *Haemophilus influenzae* type b (Hib) conjugate vaccines have reported inconsistent findings including evidence of B-cell priming [23,24] and induction of hyporesponsiveness [25], depending on the carrier protein used. Furthermore, T cell responsiveness varies within and possibly between populations as a result of variation in population genetics and environmental factors [26–31]. Therefore, before considering large-scale randomized controlled trials, it is essential to determine the safety and immunogenicity of neonatal pneumococcal conjugate vaccination in high-risk populations, including its effects on vaccine-specific T-cell responses and on the developing T cell system in general.

Here we report on the effect of a primary dose of 7vPCV vaccination at birth, followed by a dose at 1 and 2 months of age, on T cell responses at 3 months of age. This study was undertaken as part of an ongoing open randomized controlled safety and immunogenicity trial with 7vPCV in PNG. Our primary aim was to determine the effect of neonatal 7vPCV vaccination on the activation of protein carrier-specific T cell responses. Secondary aims were to evaluate potential non-specific effects on cellular immune responses to concomitant vaccines and interference with the postnatal maturation of the T cell and innate immune system.

## 2 Materials and methods

### 2.1 Study area and participants

The Asaro Valley in the Eastern Highlands Province of PNG, where this study was performed, lies 6° south of the equator and at 1500–1900 m above sea level. Goroka town is the provincial capital. The PNG Institute of Medical Research (PNGIMR) headquarters, laboratories and small clinic are located next to Goroka Hospital, the only tertiary hospital in the province. Pregnant women were recruited at Goroka Hospital antenatal clinic and also in villages located within an hour's drive of Goroka town and under monthly demographic surveillance by PNGIMR local reporters. Inclusion criteria for enrolment of newborns were the intention to remain in the study area for at least 2 years, a birth weight of at least 2000 g, no acute neonatal infection and no severe congenital abnormality. While there was no routine HIV testing, antenatal testing is recommended and children of mothers known to be HIV positive were excluded.

### 2.2 Ethical considerations

Assent was sought from women and their partners at the time of recruitment. Written informed consent was obtained after delivery and before enrolment of the newborn child. Ethical approval was obtained from the PNG Medical Research Advisory Committee and the Princess Margaret Hospital Ethics Committee in Perth, Australia.

### 2.3 Study design

Newborns were randomized using computer-generated random number lists to receive three doses of 7vPCV (Prevnar^®^, Wyeth) at birth, 1 month and 2 months of age (neonatal group) or at 1, 2 and 3 months of age (infant group), or to receive no 7vPCV (control group). Each dose of 7vPCV contained 20 μg of CRM_197_ carrier protein coupled to seven pneumococcal serotypes (4, 6B, 9V, 14, 18C, 19F, 23F) and 0.125 mg aluminium adjuvant. Laboratory staff was blinded to the randomization code.

In addition to the study vaccine, all children were to receive the immunizations recommended in PNG: Bacillus Calmette-Guérin vaccine (BCG) at birth; oral polio vaccine (OPV) at birth, 1, 2 and 3 months of age; Hepatitis B vaccine (HepB) at birth, 1 and 3 months of age; and a combined Hib, diphtheria, tetanus, whole cell pertussis vaccine (DTwP/Hib) at 1, 2 and 3 months of age. Due to a temporary shortage of BCG vaccine in PNG, BCG was given more than 72 h after birth to 30 study children (12 neonatal, 7 infant and 11 controls).

A clinical examination was done before each vaccination. Vaccination was postponed if children had a temperature > 38 °C. Research nurses visited children 48–96 h post-vaccination to interview parents and examine the children for systemic symptoms (including fever, raised respiratory rate, irritability, drowsiness, diarrhoea, vomiting and/or rash) and local reactions (tenderness, swelling and/or redness at the vaccination site). Follow-ups outside this time frame were excluded from this analysis.

### 2.4 Isolation of peripheral mononuclear cells

A venous blood sample (1–2.5 ml) was collected from children at 3 months of age into a sterile tube containing 100 IU preservative-free heparin and centrifuged within 2 h to separate plasma. Cells were resuspended in RPMI 1640 (Invitrogen-Life Technologies, Melbourne, Australia) containing preservative-free heparin (20 U/ml) and centrifuged over a Ficoll-Hypaque gradient (Lymphoprep, Alexis-Shield, Oslo, Norway) to isolate peripheral mononuclear cells (PBMC). PBMC were cryopreserved in 50% heat-inactivated foetal calf serum (HI-FCS) and 7.5% DMSO and transported to the Institute of Child Health Research (ICHR) in Perth for further analysis.

### 2.5 *In vitro* PBMC cultures

To assess T-cell responses, PBMC were resuspended in RPMI/5% heat inactivated human AB serum (Pharmacia Australia Pty. Ltd., Sydney, Australia), plated into 96-wells plates (1 × 10^6^ cells/ml) and stimulated (in duplicate) with medium (control), CRM_197_ (kindly provided by Wyeth Pharmaceuticals, USA) (2.5 μg/ml) and phytohemagglutinin (PHA; Remel Europe Ltd, Kent, UK) (1 μg/ml). When sufficient cells were available, further stimulations were performed with soluble Hepatitis B antigen (HbsAg; ProSpec-Tany TechnoGene, Rehovot, Israel) (2.5 μg/ml), mycobacterium purified protein derivative (PPD; CSL, Victoria, Australia) (10 μg/ml) and tetanus toxoid (TT; CSL, Victoria, Australia) (0.5 lf/ml). Supernatants were collected after 96 h of culture (48 h for PHA).

To measure innate immune responses, PBMC were resuspended in RPMI/10% non-heat inactivated FCS (1 × 10^6^ cells/ml) and stimulated (single wells) with medium (control), *Staphylococcus aureus*-derived lipoteichoic acid (LTA, InvivoGen, San Diego, USA) (2 μg/ml), polyinosinic–polycytidylic acid (PolyIC, Sigma, Saint Louis, USA) (50 μg/ml), *Escherichia coli*-derived lipopolysaccharide (LPS, serotype R515, Alexis Biochemicals, San Diego, USA) (5 μg/ml), and type C oligonucleotide CpG (InvivoGen, SanDiego, USA) (3 μg/ml). Supernatants were harvested after 24 h and cell pellets were stored in RNA-later (Ambion, Austin, TX, USA).

### 2.6 Cytokine protein detection

Supernatants were stored at 4 °C for a maximum of 7 days before protein levels of IL-5, IL-6, IL-10, IL-13, IFN-γ and TNF-α were measured by in-house time-resolved fluorometry [32]. Samples with concentrations below the detection limit were given the value corresponding to half the lowest detection concentration: 1.5 pg/ml for IL-5, IL-6, IL-10 and IL-13; 1.75 pg/ml for TNF-α; and 2 pg/ml for IFN-γ.

### 2.7 Quantitative (q)RT-PCR

Total RNA was isolated using the RNAqueous 96 well Kit (Ambion) according to the manufacturer's instructions. Reverse transcription was performed using the Quantitect kit (Qiagen, USA) according to the manufacturer's protocol with oligo-dT (Promega, USA) and Superasin (GeneWorks, Australia). Intron-spanning primers for IL-23p19, myxovirus resistance protein A (MxA), Granzyme B and lymphotoxin-α were obtained from http://pga.mgh.harvard.edu/primerbank and designed in-house using Primer Express Software (Applied Biosystems, USA). Reverse-transcribed RNA samples were diluted 1/5 and quantitated by real-time PCR using QuantiTect SYBR Green Master Mix (Qiagen) on the ABI PRISM 7900HT (Applied Biosystems). Copy numbers were determined by 10-fold serial dilutions of plasmid standards and normalized to the reference gene UBE2D2 [33].

### 2.8 Statistical analysis

To compare categorical variables chi-square analysis was performed and the Pearson chi-square and Cramer's *V* were calculated for 2 × 2 and 2 × 3 tables, respectively. Mann–Whitney test or Kruskal–Wallis test were used to compare continuous data in two or three groups, respectively. Cytokine responses were log_10_ transformed and data presented as geometric means (GM) ± the standard error of the geometric means (SEGM). Where indicated, *Z*-scores were calculated according to the equation: log_10(delta CRM-cytokine)_ − mean of log_10(delta CRM-cytokine)_/standard deviation of log_10(delta CRM-cytokine)_.

To study the effect of age at BCG vaccination on T cell cytokine responses at 3 months (*Z*-scores), linear regression analysis (ANOVA) was used.

Differences were considered significant if the *p*-value was smaller or equal to 0.05.

## 3 Results

### 3.1 Study population

Of the 488 pregnant women who assented, 318 newborns were enrolled and randomized to the neonatal 7vPCV group (*n* = 104), the infant 7vPCV group (*n* = 105), or the control group (*n* = 109). Within the 3 month study period, 36 children had left the study or were excluded: 1 child had died of pneumonia (infant group), 25 children were withdrawn or lost to follow up (10 neonatal, 5 infant, 10 control group), 5 children had protocol violations recorded (2 neonatal, 1 infant, 2 control group), and 5 children had a late detection of exclusion criteria including HIV and congenital heart disease (2 neonatal, 3 control group). Of the remaining 282 children sufficient PBMC for cellular immunological assays (a minimum of 1.5 × 10^6^ cells) were available for 198 children at 3 months (68 neonatal, 68 infant, 62 control group). Characteristics for these 198 newborns and their mothers are summarized in Table 1. There were no significant differences between the three study arms.

### 3.2 Reactogenicity

Children experienced few local side effects to 7vPCV vaccination (20/373, 5.4%) and few systemic side effects following any vaccination between birth and 3 months (52/705, 7.4%). For children in the neonatal group a total of six local reactions to 7vPCV were recorded (0/60 at birth, 2/62 at 1 month, 4/59 at 3 months) and for children in the infant group 14 reactions were recorded (3/63 at 1 month, and 8/64 at 2 month, and 3/64 at 3 months) (*p* = 0.084). Reactions predominantly involved tenderness of the vaccination site. Systemic side effects, which predominantly involved irritability, were recorded in 22 children in the neonatal group after vaccination at birth, 1 month, 2 months or 3 months (1/60 at birth, 9/62 at 1 month, and 7/59 at 2 months, and 5/56 at 3 months), for 16 children in the infant group (0/61 at birth, 7/63 at 1 month, 5/63 at 2 months, and 4/65 at 3 months), and 14 children in the control group (2/54 at birth, 6/55 at 1 month, 5/55 at 2 months, and 1/53 at 3 months) (*p* = 0.387).

To date there have been no serious adverse events related to the study vaccine.

### 3.3 T-cell responses to the protein carrier

**Fig. 1:**
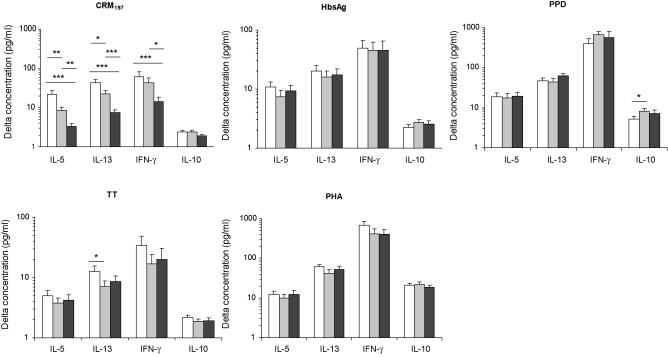
T cell responses to 7vPCV and concomitant vaccines. Peripheral blood mononuclear cells were cultured *in vitro* with medium only, or stimulated with the vaccine protein carrier CRM_197_, the polyclonal phytohemagglutinin (PHA), and vaccine antigens including soluble Hepatitis B antigen (HbsAg), mycobacterium purified protein derivative (PPD) and tetanus toxoid (TT). Antigen-specific cytokine responses were calculated by subtracting background levels that were produced in cultures with medium only from responses measured in stimulated cell cultures (‘delta concentration’). Represented are the geometric means (GMs) and standard errors of geometric means (SEGMs) for children randomized to the neonatal (white bar), infant (grey bar) and control group (black bar). Significant differences between groups are indicated at the 0.001 (***), 0.01 (**) and 0.05 (*) level.

Children in both the neonatal and infant 7vPCV groups produced significantly higher Th1 (IFN-γ) and Th2 (IL-5 and IL-13) cytokine responses at the age of 3 months in response to CRM_197_ compared to children in the control group (Fig. 1). CRM_197_ responses in the control group are most likely explained by cross-reactivity with diphtheria antigen in the DTwP/Hib vaccine. The elevated Th2 cytokine responses in the 7vPCV groups resulted from both a higher proportion of children being responders and actual higher levels that responders produced: 68% and 40% of children in the neonatal and infant group were IL5-CRM_197_ responders (as defined by levels of at least four times their background) compared to 13% in the control group (*p* < 0.001) and produced (geometric) mean levels of respectively 63.5 pg/ml (SEGM ± 8.9), 47.7 pg/ml (±7.7) and 27.2 pg/ml (±5.0) (*p* = 0.045). The same was found for IL13-CRM_197_: the proportion of positive responders (levels ≥ 4 times background) was 68% for the neonatal group, 47% for the infant group and 24% for the control group (*p* < 0.001) and produced levels (GM ± SEGM) of 94.6 pg/ml (±12.89), 68.7 pg/ml (±10.7) and 29.6 pg/ml (±4.5) respectively (*p* < 0.001). For IFNγ-CRM_197_ no differences were found for the proportion of responders (44%, 46% and 44% in the neonatal, infant and control group respectively) (*p* = 0.947), but positive responders in the neonatal (135.4 ± 49.1 pg/ml) and infant groups (91.7 ± 33.4 pg/ml) produced significantly higher levels than those in the control group (19.0 ± 5.8 pg/ml) (*p* = 0.013). The proportion of IL10-CRM_197_ responders was low and similar for the three groups: 6% in the neonatal group, 6% in the infant group and 0% in the control group (*p* = 0.150). TNF-α responses could not be detected (GM is 1.8 pg/ml for the neonatal group and equal to the cut-off value of 1.75 pg/ml for the infant and control groups), which suggests that CRM_197_ responses were mainly cognate.

IL-5 and IL-13 responses to CRM_197_ were significantly higher in the neonatal than in the infant group (*p* = 0.002 and *p* = 0.015, respectively) (Fig. 1). This was explained by significant differences in the proportion of responders (IL5, *p* = 0.001; IL-13, *p* = 0.005) and not levels these responders produced (*p* = 0.273 and *p* = 0.185, respectively). There were no differences for IFN-γ responses between the neonatal and infant group.

**Fig. 2:**
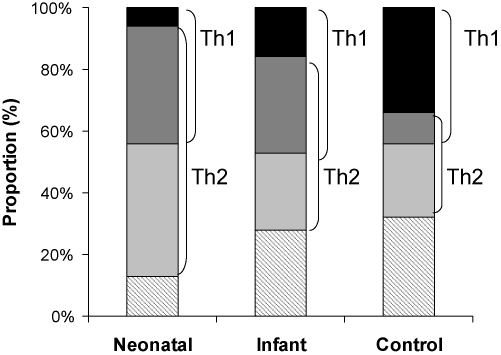
Co-production of CRM_197_-specific Th1 and Th2 responses in neonatal, infant and control groups. Children were considered to produce Th1 responses to the 7vPCV protein carrier CRM_197_ when *in vitro* CRM_197_-induced IFN-γ responses were at least four times the background level, and Th2 responses if CRM_197_-induced IL-5 and/or IL-13 responses were at least four times the background. For each study group the proportion of children with no memory responses (hatched), Th2 without Th1 (light grey), mixed Th1/Th2 (dark grey) and Th1 without Th2 (black) is presented.

As illustrated in Fig. 2, there was no difference in the proportion of Th1-CRM_197_ responders in the three groups, but in the neonatal group most of the Th1 responders (86%) produced Th2 cytokine responses (IL5-CRM_197_ and/or IL13-CRM_197_) in conjunction, whereas only 14% (6% of the total study group) produced Th1 responses ‘in isolation’ compared to 32% of the Th1 responders in the infant group (*p* = 0.072) (16% of total infant group) and 78% of the Th1 responders in the control group (34% of total control group) (*p* < 0.001).

### 3.4 Bystander effect on T-cell responses

There were no differences in Th1 or Th2 cytokine responses to PPD (BCG). Th1 and Th2 responses to HbsAg (Hepatits B), TT (DTwP/Hib) and the polyclonal PHA tended to be highest in the neonatal 7v-PCV group, but this was only significant for IL-13 responses to TT when compared to the infant group (*p* = 0.049) (Fig. 1).

### 3.5 TLR-mediated immune responses

**Fig. 3:**
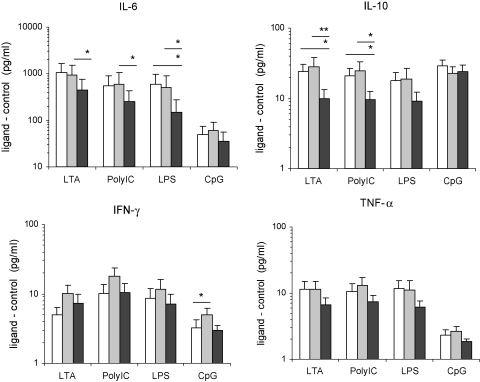
TLR-mediated immune responses. PBMC of 3-month-old children in the neonatal (white bar), infant (grey bar) and control group (black bar) were stimulated *in vitro* with lipoteichoic acid (LTA) (neonatal *n* = 37; infant *n* = 39; control *n* = 33), polyinosinic–polycytidylic acid (Poly:IC) (neonatal *n* = 40; infant *n* = 38; control *n* = 34), lipopolysaccharide (LPS) (neonatal *n* = 42; infant *n* = 40, control *n* = 35) and oligonucleotide CpG (CpG) (neonatal *n* = 32; infant *n* = 34; control *n* = 27). Bars represent geometric means and standard errors of geometric means of ligand specific cytokine responses (minus background response).

Children in the neonatal and infant 7vPCV vaccination groups produced higher IL-10 and IL-6 responses to the TLR ligands LTA (TLR2) (IL-10, *p* = 0.006; IL-6, *p* = 0.053), poly:IC (TLR3) (IL-10, *p* = 0.015; IL-6, *p* = 0.049) and LPS (TLR4) (IL-10, *p* = 0.052; IL-6, *p* = 0.028) than the control group (Fig. 3). A similar trend was seen for TNF-α responses, but differences were not statistically significant. TLR-mediated IFN-γ responses were generally higher in the infant group, but this was only significant in response to CpG.

**Fig. 4:**
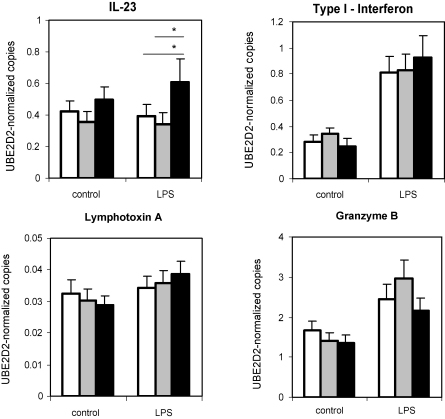
LPS-induced expression of inflammatory and cytotoxic mediators. PBMC of 3-month-old children in the neonatal 7vPCV (white bar), infant 7vPCV (grey bar) and control group (black bar) were stimulated *in vitro* with lipopolysaccharide (LPS). MRNA expression of IL-23, type-I interferon, Granzyme B and lymphotoxin-α were measured and normalized (ratio) for the expression of the housekeeping gene UBE2D2. Bars represent the geometric means and standard errors of geometric means of normalized mRNA expression in cells cultured in medium only (control) and LPS.

We further investigated the possible modulation of other inflammatory mediators including type-I interferon, IL-23, Granzyme B and lymphotoxin-α in response to LPS stimulation, but other than a reduced expression of IL-23 in the 7vPCV vaccination groups, no differences were found (Fig. 4). All three groups showed an enhanced release of mature IL-1β in response to LPS stimulation, but there were no significant differences between the groups (neonatal 315.5 ± 101.4 pg/ml, infant 334.1 ± 163.9 pg/ml, 303.6 ± 103.7 pg/ml) (*p* = 0.955).

### 3.6 Effect of BCG immunization

BCG vaccination was delayed (more than 3 days after birth) in 30 study children (12 neonatal, 7 infant and 11 controls). Although significant associations were found between PPD-specific IL-13 responses at 3 months and the age at which children had received BCG, there were no significant effects with respect to cytokine responses to CRM_197_ (Table 2), HbsAg or PHA (data not shown), either overall or when the neonatal group was examined separately (data not shown).

## 4 Discussion

To our knowledge this study in PNG is the first safety and immunogenicity trial of 7vPCV in human newborns to include an evaluation of cellular immune responses. We have found that neonatal 7vPCV immunization is well tolerated, does not induce tolerance but rather primes carrier protein-specific T-cell responses with a preferential expression of Th2 cytokines, and has no detrimental effects on general T cell ontogeny.

In line with 7vPCV studies involving adults and toddlers in the USA [13,14,34], carrier protein-specific T-cell responses in 3-month-old PNG children were of a mixed Th1/Th2 phenotype. However, compared to children immunized with 7vPCV at 1 and 2 months of age, children primed with a neonatal dose of 7vPCV produced significantly higher Th2, but not Th1, cytokine responses to CRM_197_. Moreover, children in the neonatal group generally produced Th1 cytokine responses in conjunction with Th2, and not ‘in isolation’ as was more often the case for the infant group. These findings suggest that neonatal 7vPCV vaccination stimulates Th2 responses, whereas Th1 responses may be activated following immunizations at 1 and 2 months of age. We can conclude that, in contrast to neonatal BCG vaccination, a neonatal dose of 7vPCV does not have the capacity to stimulate neonatal T-cells to generate significant Th1 responses [21,22], at least not in this study population.

Variation in the Th1/Th2 response may influence the antibody isotype and priming for B cell memory of vaccine-related pneumococcal serotypes [12–14], but how this affects protection is not clear. In neonatal mice impaired Th2 cytokine responses have been directly related to reduced antibody responses and protection against pneumococcal infections, whilst infant mice producing Th2 but no Th1 responses were able to resist challenges [20]. Thus despite the inability to prime Th1 responses, the capacity of neonatal 7vPCV vaccination to activate specific Th2 responses in human newborns could be sufficient to stimulate functional antibody responses and provide protection against invasive disease in the first weeks of life. The associations between neonatal 7vPCV-induced Th2 cytokine responses, antibody responses to 7vPCV serotypes and risk of carriage and disease will be addressed in our ongoing studies.

The skewing of Th2 responses following neonatal 7vPCV immunization may be relevant to subsequent local reactions to 7vPCV or DTP booster immunizations. We have previously shown that Th2 skewing of cytokine responses to tetanus toxoid in association with vaccine specific IgE was correlated with large local reactions to diphtheria-tetanus-acellular pertussis boosters at 4 years of age [35]. In PNG this may be less relevant as DTwP is used and booster immunizations are not given to older children. Moreover, reactogenicity to 7vPCV immunization was low in both study groups, in particular in the neonatal group, which provides reassurance for the safety of 7vPCV immunization at birth.

CRM_197_ is the product of a single missense mutation of diphtheria toxin (DT) that attenuates its toxicity but not immunogenicity. Immune responses to DT and CRM_197_ are therefore similar, but in 7vPCV the immunogenicity of CRM_197_ may be reduced due to conjugation of pneumococcal polysaccharides [12]. Although the capacity of 7vPCV to induce memory T-cell responses in PNG newborns and infants is obvious from the significantly elevated CRM_197_ responses compared to the control group, based on this study it cannot be determined whether immunization with 7vPCV has an additive, synergistic or suppressing effect on DT responses induced by DTwP-Hib.

Although immunization with 7vPCV did not interfere with T cell responses to co-administered vaccines or with the maturation of T cell responses in general, TLR-mediated responses were modulated as shown by enhanced IL-6 and IL-10 responses in both 7vPCV vaccination groups. One possible explanation may be the presence of aluminium adjuvant (Alum) in the study vaccine. Although all children receive Alum in the DTwP/Hib vaccine, this is as a hydroxide salt rather than phosphate as is used in the 7vPCV. Recent studies have shown that Alum induces the release of mature IL-1β [36,37], which can stimulate IL-6 and IL-10 production [37–39], and may have a long-lasting and non-localized effect through circulation of inflammatory dendritic cells [37,40] that predominantly induce Th2 responses [37,41]. Although we were not able to demonstrate enhanced IL-1β responses in the 7vPCV vaccinated children, this may be explained by findings that concomitant TLR and Alum stimulation is required to induce IL-1β secretion *in vitro* [36]. The persistence of this modulated innate immune response and the potential effects of IL-10 and IL-6 on promoting tolerance [42,43] and Th17 development [44–46] will be the subject of future investigations when the study children are older.

In summary, in this study we have demonstrated that neonatal 7vPCV immunization primes carrier protein-specific Th2-associated responses in PNG infants. The power of this study is that rather than studying non-specific immune responses that are a net outcome of a plethora of exposures to infections, environmental antigens and other vaccinations, antigen-specific responses were analysed and bystander effects were investigated. Accelerated 7vPCV vaccination had no detectable bystander effects on T cell responses, but significantly enhanced TLR-mediated IL-6 and IL-10 responses. The potential implications of these skewed Th2 and innate immune responses on subsequent immune responses up to age 18 months are the subject of ongoing studies.

## Figures and Tables

**Table 1:** Population description.

	Neonatal (*n* = 68)	Infant (*n* = 68)	Control (*n* = 62)	*p*-Value
Mother characteristics				
Mean age (years)	25.8 (5.4)	25.6 (5.3)	25.2 (5.7)	0.715
Gravida: 1	27%	30%	37%	
Gravida: 2	35%	21%	28%	
Gravida: 3	21%	21%	15%	
Gravida: 4+	17%	27%	20%	0.468
Delivery by caesarean	3%	2%	2%	0.807
Smoking in pregnancy	8%	7%	15%	0.251
Tetanus vaccination	93%	88%	93%	0.473

Newborn characteristics				
Male	44%	54%	55%	0.373
Mean gestational age (weeks)	39.4 (1.3)	39.7 (1.2)	39.7 (0.2)	0.299
Mean birth weight (kg)	3.2 (0.4)	3.3 (0.4)	3.2 (0.5)	0.457
Mean birth length (cm)	49.7 (3.2)	49.8 (3.8)	50.5 (3.2)	0.459
Mean head circumference (cm)	33.2 (1.4)	33.3 (1.8)	33.3 (1.5)	0.872
Mean weight at age 3 months (kg)	6.3 (0.8)	6.2 (0.9)	6.4 (0.7)	0.358

Mean age at vaccination, days				
BCG	9.8 (30.1)	3.4 (7.6)	2.7 (4.9)	0.780
7vPCV – 1	1.3 (0.9)	30.0 (2.8)	N/A	N/A
7vPCV – 2	30.2 (2.8)	61.1 (5.5)	N/A	N/A
7vPCV – 3	62.5 (5.8)	92.7 (7.1)	N/A	N/A
Hepatitis B – 1	0.9 (1.0)	1.2 (1.8)	1.2 (1.3)	0.547
Hepatitis B – 2	30.2 (2.8)	30.0 (2.8)	30.4 (4.9)	0.684
Hepatitis B – 3	93.5 (9.3)	92.7 (7.1)	90.1 (7.3)	0.185
OPV – 1	2.2 (2.6)	3.9 (8.7)	4.4 (10.2)	0.169
OPV – 2	31.7 (6.9)	33.2 (10.8)	32.2 (8.3)	0.774
OPV – 3	63.2 (7.4)	63.4 (12.0)	62.9 (8.3)	0.500
OPV – 4	94.7 (9.3)	92.0 (6.6)	94.1 (17.3)	0.301
DTwP/Hib – 1	30.1 (3.3)	30.0 (2.8)	30.4 (4.9)	0.723
DTwP/Hib – 2	62.4 (5.7)	61.1 (5.5)	61.5 (6.4)	0.398
DTwP/Hib – 3	93.5 (9.3)	92.7 (7.1)	92.8 (16.3)	0.406

Continuous data are represented as mean (standard deviation). BCG, Bacillus Calmette-Guerin vaccine; 7vPCV, 7-valent pneumococcal conjugate vaccine; OPV, oral polio vaccine; DTwP/Hib, combined *Haemophilus influenzae* type b diphtheria, tetanus, whole cell pertussis vaccine.

**Table 2:** Effect of age at BCG vaccination on cytokine responses at 3 months.

	CRM_197_		PPD	
	Coefficient *β* (95% CI)	*p*-Value	Coefficient *β* (95% CI)	*p*-Value
IL-5	0.003 (−0.018 to 0.023)	0.808	−0.017 (−0.039 to −0.004)	0.114
IL-13	0.001 (−0.019 to 0.021)	0.928	−0.023 (−0.044 to −0.002)	0.033
IFN-γ	− 0.001 (−0.021 to 0.020)	0.940	−0.016 (−0.037 to 0.005)	0.138
IL-10	− 0.002 (−0.023 to 0.018)	0.815	−0.013 (−0.035 to 0.009)	0.255

Linear regression analysis was used to study associations between cytokine responses to the 7vPCV protein carrier CRM_197_ and PPD in study children when they were 3 months old and their age when they were vaccinated with BCG (independent variable).
